# Estimation of the External Knee Adduction Moment Using Inertial Measurement Unit Sensors on the Shank and Lower Back: A Pilot Study

**DOI:** 10.3390/jfmk10030356

**Published:** 2025-09-18

**Authors:** Tomoaki Matsuda, Junichi Watanabe, Tasuku Sotokawa, Toru Shishime, Hiroshi Katoh

**Affiliations:** 1 Department of Rehabilitation, Shishime Orthopedic Hospital, Miyazaki 880-0121, Japan; hqmcb719@gmail.com; 2 Graduate School of Health Sciences, Yamagata Prefectural University of Health Sciences, Yamagata 990-2212, Japan; 3 Department of Orthopedic Surgery, Shishime Orthopedic Hospital, Miyazaki 880-0121, Japan

**Keywords:** external knee adduction moment, IMU sensor, waveform pattern, gait, early phase of single-limb support

## Abstract

**Background:** The external knee adduction moment (KAM) is an important biomechanical parameter that reflects the load on the medial tibiofemoral compartment during gait. The KAM is typically evaluated using three-dimensional motion analysis (3DMA) systems. The present study aimed to evaluate and validate the waveform similarity between the KAM estimated using only two inertial measurement units (IMUs) sensors, attached to the shank and lower back (IMU-KAM), as a simpler method and that obtained from a 3DMA system (3DMA-KAM) under different step rate conditions. **Methods:** Three healthy adult men were included. The gait task involved walking in a straight line over a distance of approximately 10 m at three step rate conditions: 115, 100, and 85 steps/min. Data were collected using a 3DMA system, force plates, and IMUs. The primary outcome measures included the KAM waveforms for 3DMA-KAM and IMU-KAM during the early and late phases of the single-limb support (Early-SLS phase and Late-SLS phase, respectively). The coefficient of multiple correlation (CMC) was used to evaluate the waveform pattern similarity. **Results:** IMU-KAM demonstrated high similarity to 3DMA-KAM waveforms in the Early-SLS phase under 115 and 100 steps/min, with CMC values ranging from 0.66 to 0.99. However, no clear similarity was observed in the Late-SLS phase. **Conclusions:** In the Preferred and Reduced conditions, wherein the walking rate exceeded 100 steps/min, the KAM waveform pattern during the Early-SLS phase was accurately estimated using IMU sensors attached to the shank and lower back. The findings of this study suggest the potential of simplified gait analysis using IMUs for evaluating knee joint biomechanics and provide foundational data for future clinical applications.

## 1. Introduction

Gait analysis is a widely used and effective method for evaluating motor function and the effects of interventions in the field of rehabilitation [1,2,3,4]. Hulleck et al. [1] reviewed various gait analysis methods and their clinical applications, including the identification and quantification of disease-specific gait impairments and the prediction of prognosis. Wren et al. [2] performed a systematic review on the clinical utility of gait analysis using measurement devices and reported that it can inform treatment decision-making and determine therapeutic effects. Furthermore, Miyashita et al. [3] suggested the potential of detecting gait impairments using a single inertial measurement unit (IMU) sensor in older adults at an early stage. Boyer et al. [4] also conducted a systematic review and meta-analysis involving both young and older adults and clarified age-related biomechanical changes during gait. In gait biomechanics studies, the estimation of joint loading is a key research topic, and its significance is particularly emphasized in the gait analysis of patients with knee osteoarthritis (OA). Among those changes, the external knee adduction moment (KAM) has attracted attention as an indicator of the load on the medial tibiofemoral compartment and has been reported as a vital biomechanical parameter [5,6,7,8,9]. During gait, the KAM waveform generally exhibits a bimodal pattern, with the first peak occurring in the early phase of single-limb support (SLS) after the initial contact and the subsequent reduction during midstance [10,11,12,13]. Previous studies considered both features clinically important indicators due to their association with the onset, progression, and severity of knee OA [10,11,12,13]. Three-dimensional motion analysis (3DMA) based on optical motion capture systems combined with force plates is the gold standard for gait analysis using KAM [1,8,14,15,16]. 3DMA enables high-precision calculation of KAM by combining joint center positions and joint angles derived from three-dimensional coordinate data obtained via optical motion capture with three-dimensional ground reaction forces directly measured using a force plate. However, these systems are expensive and require a dedicated laboratory environment, limiting their clinical application [15,16,17]. To address these limitations, recent studies estimated the KAM using inertial measurement units (IMUs) [16,18,19,20]. Iwama et al. [16] demonstrated the potential for simplified KAM estimation by showing a correlation between acceleration-based indices from a single IMU sensor and the first peak of KAM measured using 3DMA. Stetter et al. [18] reported estimation methods applicable to various locomotor tasks by combining IMU sensors on the thigh and shank with machine learning. Moreover, Akiba et al. [19] applied machine learning and a proprietary algorithm to a single IMU sensor and reported that KAM during gait can be estimated with clinically practical accuracy. Kobsar et al. [20] also reviewed multiple approaches, including the use of multiple IMU sensors combined with instrumented force shoes and methods using IMU sensors attached to the foot for estimating KAM. Nevertheless, most of these methods involve constructing full-body skeletal models with multiple IMUs or require machine learning algorithms.

By contrast, Baniasad et al. [21] investigated the relationship between the KAM and four important gait elements (i.e., ground reaction force [GRF], shank tilt angle [STA], foot progression angle [FPA], and center of pressure [COP]). The results showed that the vertical component of the GRF (GRF-VT) and frontal plane STA (FP-STA) were the most influential factors contributing to increased KAM in individuals with knee OA. Importantly, these two parameters can be estimated using IMUs, suggesting that IMU-derived data can be used to estimate the KAM [22,23,24,25,26,27,28].

Moreover, previous studies have shown that the peak value and waveform pattern of KAM are influenced by spatiotemporal gait parameters, including walking speed and step rate [29,30,31]. In particular, the step rate has been reported to exert a remarkable effect on the KAM [29,30,31], indicating that step rate variations need to be considered when estimating the KAM.

Therefore, the present study was conducted to investigate and validate the waveform similarity between KAM estimated using only two IMU sensors, attached to the shank and lower back (IMU-KAM), as a simpler method and KAM obtained from a 3DMA system (3DMA-KAM) under different step rate conditions. Based on previous findings indicating that the first KAM peak is primarily influenced by the GRF-VT and knee adduction angle [32], we hypothesized that the KAM waveform during the early phase of SLS could be estimated with clinically acceptable accuracy.

## 2. Methods

### 2.1. Participants

This study included three healthy adult men: participant 1 (age, 21 years; body height, 172.0 cm; body weight, 64.0 kg), participant 2 (age, 24 years; body height, 170.5 cm; body weight, 67.3 kg), and participant 3 (age, 46 years; body height, 171.0 cm; body weight, 63.0 kg). The inclusion criterion was as follows: absence of any neurological, psychiatric, or orthopedic disorders. Meanwhile, the exclusion criterion was as follows: presence of any pain during walking.

Before the study, approval was obtained from the ethics committee of our institution (Approval No. 2409-01). All participants were provided with a detailed explanation of the study’s purpose and significance, and written informed consent was obtained.

### 2.2. Gait Task

The participants walked in a straight line over a distance of approximately 10 m under three step rate conditions. The step rate was controlled using a metronome and set at 115 steps/min (preferred step rate: Preferred), 100 steps/min (reduced step rate: Reduced), and 85 steps/min (lowest step rate: Lowest). No restrictions were imposed on the step length. The step rate conditions were based on previous studies [33,34,35,36,37]. The gait task was performed in the following fixed order: Preferred, Reduced, and Lowest. Two trials were performed for each condition, yielding a total of six trials per participant. The changes in step rate were manually implemented for each condition.

### 2.3. Data Collection

An optical motion capture system (optical system) was used for the measurements. This system comprised a 3DMA device (VICON MX-T, VICON Motion Systems Ltd., Oxford, UK) with 16 cameras and six force plates (OR6-7-2000, Advanced Mechanical Technology Inc., Watertown, MA, USA), as shown in Figure 1.

In addition, an inertial motion capture system (inertial system) comprising two IMU sensors (SS-MS-HMA5G3A2YZ, Sports Sensing Inc., Fukuoka, Japan) and one wireless synchronization unit (Sports Sensing Inc., Fukuoka, Japan), which was synchronized with the optical system, was used.

During the measurements using the optical system, a seven-segment rigid body model, which comprised the foot, shank, thigh, and pelvis segments, was constructed by attaching 33 infrared reflective markers (14 mm in diameter) to the participant’s body, in accordance with the method of Kito et al. [38]. For the inertial system, two sensors were attached: one to the spinous process of the third lumbar vertebra (L3) and the other to the lower lateral shank (LLS) of the right leg [39]. The axes of both IMU sensors were aligned as closely as possible with the global coordinate system. The L3 IMU sensor was oriented with the X-, Y-, and Z-axes in the mediolateral, vertical, and anteroposterior direction, respectively. Conversely, the LLS IMU sensor was oriented with the X-, Y-, and Z-axes in the anteroposterior, vertical, and mediolateral direction, respectively (Figure 2). The sampling frequency of the 3DMA device and force plates was set to 100 Hz, whereas that of the IMU sensors was set to 200 Hz.

### 2.4. Data Analysis

The primary outcome measures included the 3DMA-KAM and the IMU-KAM of the right lower limb. Meanwhile, the secondary outcome measures included the GRF-VT and the FP-STA of the right lower limb, which were calculated using the optical and inertial systems, respectively. Before data processing, a low-pass Butterworth filter was used to reduce noise. For 3DMA data, the cutoff frequencies were set at 6 and 10 Hz for the marker coordinate data and GRF data, respectively. Meanwhile, for the IMU data, a cutoff frequency of 10 Hz was applied to the vertical component of the acceleration data. This value was based on the report by Jiang et al., which indicated that most of the GRF signal lies below 10 Hz [40]. A cutoff frequency of 12 Hz was applied to the angular velocity data. This value was based on the report by Tadano et al., which provided preliminary validation using quaternions to reduce integration errors in angle estimation [41].

#### 2.4.1. Optical System

The 3DMA-KAM and center of mass were calculated using Vicon Nexus 2.12 (Vicon Motion Systems Ltd., Oxford, UK). Next, the walking speed, step length, and step rate were calculated from the center of mass, right heel marker, and GRF-VT.

#### 2.4.2. Inertial System

The calculation of IMU-KAM was conducted in accordance with the method of Baniasad et al. [21] (Figure 3). In this approach, the GRF-VT was estimated from the L3 IMU sensor, whereas the FP-STA was derived from the LLS IMU sensor. First, the inclination angles of both IMU sensors relative to the global coordinate system were calculated using data collected during quiet standing on the basis of a previous study [41]. Then, the GRF-VT was obtained by converting the vertical axis component from the inclination angles using trigonometric functions and multiplying it by body weight (N). Meanwhile, the FP-STA was defined as the frontal plane tilt angle of the LLS IMU sensor, which was adjusted at the initial value to match the STA calculated from the 3DMA system. Finally, IMU-KAM was estimated as the product of the GRF-VT and the lever arm derived from FP-STA (labeled as “Lever Arm” in Figure 3). The lever arm was calculated using trigonometric functions (sine) based on FP-STA and the shank length obtained from 3DMA [21].

#### 2.4.3. Analysis Interval

**Figure 1:**
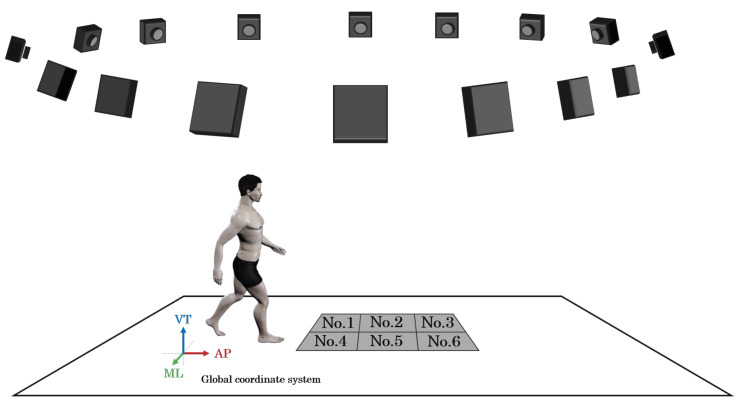
Measurement environment.

**Figure 2:**
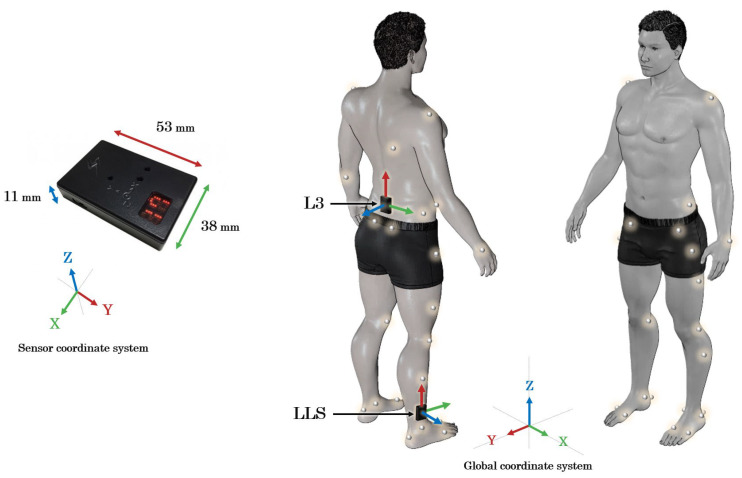
Placement of the infrared reflective markers and IMU sensors. The IMU sensor, which measured 53 mm in length, 38 mm in width, and 11 mm in thickness, equipped with an eight-axis sensor system comprising a three-axis accelerometer (±16 G), a two-axis accelerometer (±200 G), and a three-axis gyroscope (±1500 deg/s). The global coordinate system was defined as follows: X-axis in the mediolateral direction; Y-axis in the anteroposterior direction; and Z-axis in the vertical direction. L3: spinous process of the third lumbar vertebra; LLS: lower lateral shank (7 cm proximal to the lateral malleolus).

**Figure 3:**
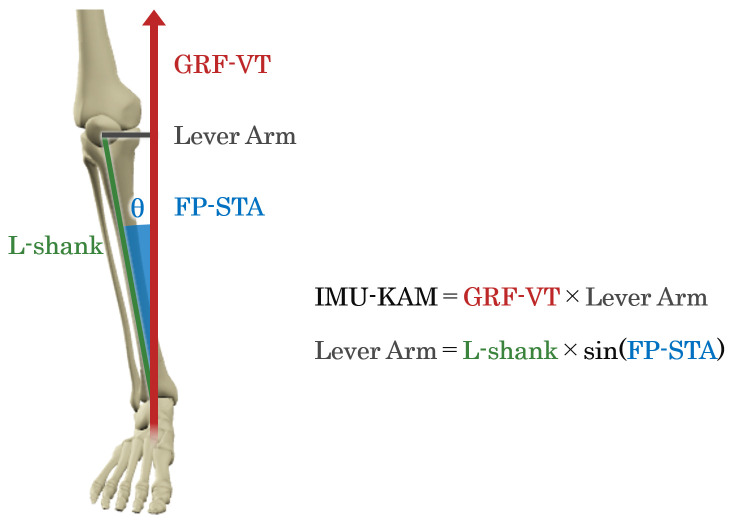
Method for estimating IMU-KAM based on the GRF-VT and FP-STA. IMU-KAM: knee adduction moment estimated using inertial measurement units (IMUs); GRF-VT: vertical component of the ground reaction force (GRF); FP-SPA: frontal plane (FP) shank tilt angle (STA); L-shank: shank length.

The analysis interval was defined as the SLS phase of the right lower limb. In this study, a single SLS phase was analyzed per trial. Specifically, the left lower limb toe-off, right lower limb stance phase, and left lower limb initial contact were identified using force plate Nos. 1, 5, and 3, respectively (Figure 1). The SLS phase was identified as the period from the point at which the GRF-VT on the left leg dropped below 10 N to just before it rose above 10 N again. This interval was time-normalized to 100%. Moreover, the SLS phase was further divided into two segments (i.e., early phase of SLS [Early-SLS phase] and late phase of SLS [Late-SLS phase]) in accordance with the method of Thorp et al. [42].

### 2.5. Statistical Analysis

The similarity of the waveform patterns between IMU-KAM and 3DMA-KAM was assessed using the coefficient of multiple correlation (CMC) [43,44,45]. Meanwhile, the similarity of the waveform patterns between GRF-VT and FP-STA estimated using IMU sensors and those measured by 3DMA was examined using CMC. The similarity was categorized into four levels based on the CMC values: moderate (0.65–0.75), good (0.75–0.85), very good (0.85–0.95), and excellent (0.95–1.00) [45].

## 3. Results

### 3.1. Walking Speed, Step Length, and Step Rate Under Different Gait Conditions

The walking speed ranged from 1.02 m/s to 1.30 m/s, 0.88 m/s to 1.08 m/s, and 0.77 m/s to 0.85 m/s under the Preferred, Reduced, and Lowest conditions, respectively. The step length ranged from 0.56 m to 0.69 m, 0.56 m to 0.65 m, and 0.56 m to 0.62 m, respectively. The step rate ranged from 111.11 steps/min to 120.00 steps/min, 100.00 steps/min to 107.14 steps/min, and 83.33 steps/min to 90.91 steps/min, respectively.

### 3.2. IMU-KAM and 3DMA-KAM

**Figure 4:**
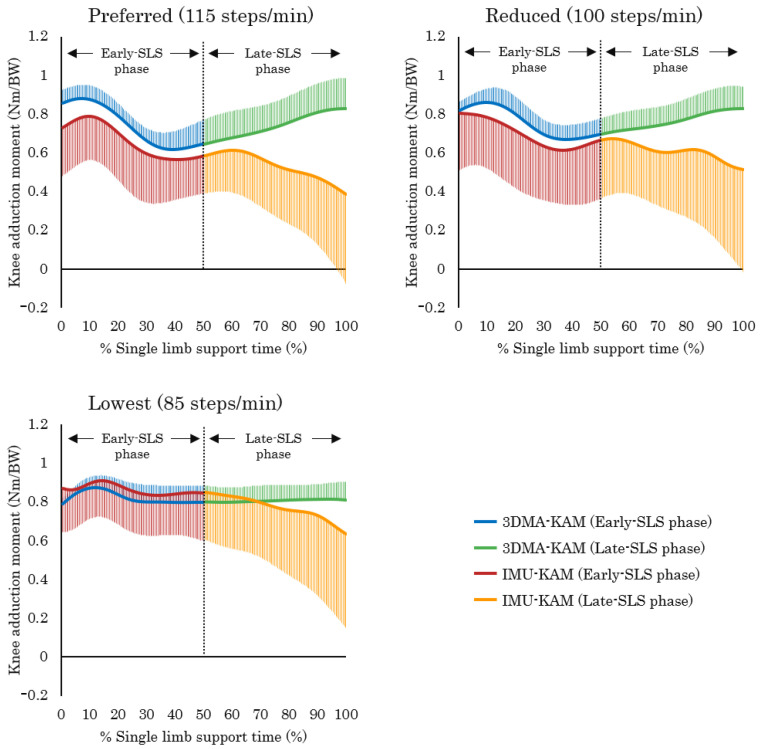
Mean and standard deviation of waveform patterns under each condition. Preferred: preferred step rate (115 steps/min); Reduced: reduced step rate (100 steps/min); Lowest: lowest step rate (85 steps/min); Early-SLS phase: early phase of single-limb support; Late-SLS phase: late phase of single-limb support; 3DMA-KAM: knee adduction moment calculated from three-dimensional motion analysis; IMU-KAM: knee adduction moment estimated using inertial measurement units (IMUs).

The mean and standard deviation of the IMU-KAM and 3DMA-KAM waveform patterns under each condition are shown in Figure 4, and the CMC values between the IMU-KAM and 3DMA-KAM during Early-SLS phase and Late-SLS phase is shown in Table 1.

#### 3.2.1. Early-SLS Phase

Under the Preferred condition, the CMC values between the IMU-KAM and 3DMA-KAM ranged from 0.67 to 0.99, indicating moderate to excellent similarity. In the Reduced condition, the CMC values ranged from 0.66 to 0.89, indicating moderate to very good similarity. Meanwhile, under the Lowest condition, two trials showed CMC values of 0.80 and 0.82, indicating good similarity. However, the values were below 0.65 in the remaining four trials, indicating no clear similarity in waveform patterns.

#### 3.2.2. Late-SLS Phase

Under the Preferred condition, four trials resulted in complex numbers, represented as not a number (NaN), and the remaining two trials yielded CMC values below 0.65, indicating no clear waveform similarity. In the Reduced condition, all trials produced NaN values. Meanwhile, under the Lowest condition, only one trial yielded a CMC value of 0.77, indicating good similarity, whereas the remaining five trials showed no evident waveform similarity.

### 3.3. GRF-VT and FP-STA

For GRF-VT, the CMC during the Early-SLS phase under the Preferred and Reduced conditions ranged from 0.67 to 0.99, with most trials rated as excellent (Table 2). Meanwhile, for the Early-SLS phase under the Lowest condition and Late-SLS phase across all conditions, some trials showed CMC values below 0.65. Moreover, no clear waveform similarity was observed. For FP-STA, the CMC values were below 0.65 in most trials across all conditions, indicating no clear waveform similarity (Table 3).

## 4. Discussion

### 4.1. Changes in Spatiotemporal Parameters Under Different Gait Conditions

Walking speed tended to vary according to the step rate conditions. Although step length varied slightly among participants, it exhibited little change across conditions. Step rate was generally similar to the values set for each condition. In this context, Sekiya et al. [46] examined how changing walking speed, step length, or step rate individually affects the other two parameters. They reported that when only step rate was altered, walking speed changed accordingly, whereas step length remained largely unchanged. These findings support the validity of the spatiotemporal changes observed under the conditions set in the present study.

### 4.2. Characteristics of IMU-KAM and 3DMA-KAM

This study examined the similarity between the KAM waveform patterns estimated from IMU sensors attached to the shank and lower back and those obtained from 3DMA measurements, with the SLS phase divided into the Early-SLS phase and Late-SLS phase. The hypothesis was that KAM estimation using IMU sensors could be achieved with clinically applicable accuracy during the Early-SLS phase. Our results supported the hypothesis under the condition of ≥100 steps/min but not under the condition of a low step rate, such as 85 steps/min.

#### 4.2.1. Characteristics of the Early-SLS Phase

This study considered the influence of the step rate on the KAM waveform patterns by analyzing three walking conditions. No clear similarity in the KAM waveform patterns was observed under the Lowest condition. KAM typically exhibits a bimodal waveform pattern; however, studies have reported that reducing the step rate or walking speed alters KAM to a unimodal pattern [31,47]. In our study, it cannot be ruled out that such changes in the waveform pattern may have affected the accuracy of KAM estimation. In patients with knee OA, lower step rates are reportedly associated with greater radiographic severity [48,49,50]. Consistent with this finding, a recent large-scale cohort study of 1600 participants grouped by step rate found that approximately 25% of individuals, classified in the lowest group, had a mean step rate of 97.6 ± 4.6 steps/min [51]. On the basis of the findings of the present study, the proposed method may be applicable for estimating the KAM waveform patterns when the step rate is 100 steps/min or higher. Therefore, this method may be applicable to the majority of patients with knee OA, except for cases with extremely reduced step rates.

With regard to the estimation accuracy of the KAM during gait, previous studies have reported Pearson’s correlation coefficients of 0.71 for waveform patterns during the stance phase, 0.58 for peak values, and 0.69 for impulse [18,19]. Existing guidelines for interpreting correlation coefficients categorize the correlation as weak (r ≤ 0.35), moderate (0.35 < r ≤ 0.67), strong (0.67 < r ≤ 0.90), and excellent (r > 0.90) [18]. On the basis of this classification, the strength of the correlations determined in our study can be interpreted as ranging from moderate to strong. Although the proposed method estimated the KAM only in the limited interval of the Early-SLS phase, its estimation accuracy was better compared with those of previous reports.

#### 4.2.2. Characteristics of the Late-SLS Phase

In the Late-SLS phase, little to no similarity in the KAM waveform patterns was observed across nearly all trials under all conditions. One possible reason is that factors that were not considered in the present method (e.g., COP and FPA) may influence the second peak of the KAM, which generally occurs during this phase [21,47,52]. Baniasad et al. [21] reported that the anterior–posterior position of COP and FPA particularly affects the second peak of KAM in patients with knee OA. Furthermore, Kim et al. [52] demonstrated that experimentally altering FPA in patients with knee OA modifies the second peak of KAM. Moreover, Rutherford et al. [47] conducted a cross-sectional study and reported that FPA was associated with the second peak of KAM, whereas its effect on the first peak was minimal. Moreover, previous studies on the KAM estimation accuracy have reported reduced accuracy during the late stance phase, corresponding to the Late-SLS phase, compared with the early and mid-stance phases. This reduction may result from the increased difficulty in estimating the ankle joint position and orientation during this period [53,54]. During the terminal stance phase, the COP shifts toward the forefoot as the ankle transitions from dorsiflexion to plantarflexion, accompanied by subtle inversion and eversion of the foot [55,56]. This complex three-dimensional motion likely contributes to decreased accuracy in estimating KAM during the terminal stance, although other factors cannot be ruled out.

### 4.3. Characteristics of GRF-VT and FP-STA

In the present study, the waveform similarity of GRF-VT and FP-STA was examined using CMC to identify the factors that contribute to the estimation accuracy of IMU-KAM. High CMC values were obtained for GRF-VT during the Early-SLS phase under both the Preferred and Reduced conditions. However, in the Early-SLS phase under the Lowest condition, CMC tended to be lower than that under the other two conditions. We consider that this tendency is consistent with the degree of waveform similarity observed in KAM. Although studies have reported that GRF-VT can be estimated with high accuracy using IMUs [25,26,27,28], considering that a similar trend in estimation accuracy was observed for both KAM and GRF-VT under the Lowest condition, it can be inferred that GRF-VT may contribute more substantially to the estimation of the KAM waveform pattern in the Early-SLS phase. In contrast, for FP-STA, many of the CMC values resulted in NaN, and no reliable consistency could be obtained. Regarding the estimation of joint angle waveform patterns, it has been reported that accuracy is high in the sagittal plane but lower in the frontal plane [57,58]. We also consider that this tendency was more pronounced in our study. Regarding the Late-SLS phase, our results suggest the difficulty of estimating accurate KAM waveforms using only GRF-VT and FP-STA information, as used in our study, indicating a potential limitation. Therefore, future studies must incorporate other related factors, such as COP and FPA, to enable a more comprehensive and multifactorial approach.

### 4.4. Strengths, Limitations, Future Directions, and Practical Applications

The strength of this study lies in the ability to accurately estimate the waveform pattern of KAM during the Early-SLS phase using only two IMU sensors, without the need for machine learning. A limitation of this study is the small sample size, due to which statistical validation was difficult. In particular, the KAM waveform pattern during the Late-SLS phase could not be clarified in detail, possibly due to the limited number of participants. Moreover, the ages of three participants were variable, resulting in an insufficient evaluation of age-related effects. Future studies should ensure an adequate sample size for each age group to allow systematic evaluation.

Regarding future directions, although this study estimated KAM using two parameters, GRF-VT and FP-STA, the agreement of the FP-STA waveform pattern was insufficient. Therefore, other relevant gait parameters should be incorporated in the analysis to improve the accuracy of estimation. Furthermore, because the present study manipulated only the step rate, considering additional factors such as step length and walking speed may further improve the accuracy and applicability of the estimation. It is also necessary to investigate other mechanical parameters, such as the external knee flexion moment.

Overall, the method described in our study can estimate KAM waveform patterns with clinically acceptable accuracy using a minimal number of IMU sensors, provided that the analysis is limited to the Early-SLS phase, thereby suggesting potential applications in clinical gait analysis and the evaluation of treatment effects.

## 5. Conclusions

This study examined whether IMU-KAM estimated using IMU sensors on the shank and lower back exhibits similar waveform patterns to those of 3DMA-KAM. The results showed that the waveform similarity was high during the Early-SLS phase under conditions with a step rate of 100 steps/min or higher, suggesting the utility of simple IMU-based estimation. By contrast, the waveform agreement decreased during the Late-SLS phase or when the step rate decreased, indicating limitations in estimation accuracy.

## Figures and Tables

**Table 1:** CMC values between IMU-KAM and 3DMA-KAM waveform patterns.

		CMC					
		Preferred		Reduced		Lowest	
		Early-SLS Phase	Late-SLS Phase	Early-SLS Phase	Late-SLS Phase	Early-SLS Phase	Late-SLS Phase
Participant 1	Trial 1	0.98	NaN	0.74	NaN	0.62	0.77
	Trial 2	0.99	0.29	0.86	NaN	0.80	NaN
Participant 2	Trial 1	0.97	0.55	0.83	NaN	0.82	NaN
	Trial 2	0.67	NaN	0.89	NaN	0.43	0.23
Participant 3	Trial 1	0.83	NaN	0.66	NaN	0.52	NaN
	Trial 2	0.93	NaN	0.75	NaN	0.48	NaN

IMU-KAM: knee adduction moment estimated using inertial measurement units (IMUs); 3DMA-KAM: knee adduction moment calculated from three-dimensional motion analysis; CMC: coefficient of multiple correlation; Preferred: preferred step rate (115 steps/min); Reduced: reduced step rate (100 steps/min); Lowest: lowest step rate (85 steps/min); Early-SLS phase: early phase of single-limb support; Late-SLS phase: late phase of single-limb support; NaN: not a number (indicates that the CMC is a complex number).

**Table 2:** CMC values between GRF-VT estimated using IMU and GRF-VT measured using 3DMA.

		CMC					
		Preferred		Reduced		Lowest	
		Early-SLS Phase	Late-SLS Phase	Early-SLS Phase	Late-SLS Phase	Early-SLS Phase	Late-SLS Phase
Participant 1	Trial 1	0.85	0.74	0.91	0.89	0.68	0.97
	Trial 2	0.99	0.94	0.93	0.92	0.46	0.69
Participant 2	Trial 1	0.97	0.89	0.91	0.57	NaN	NaN
	Trial 2	0.99	0.82	0.85	NaN	0.54	NaN
Participant 3	Trial 1	0.77	NaN	0.86	0.77	0.04	0.71
	Trial 2	0.67	0.13	0.84	0.44	0.84	0.81

GRF-VT: vertical component of the ground reaction force; CMC: coefficient of multiple correlation; Preferred: preferred step rate (115 steps/min); Reduced: reduced step rate (100 steps/min); Lowest: lowest step rate (85 steps/min); Early-SLS phase: early phase of single-limb support; Late-SLS phase: late phase of single-limb support; NaN: not a number (indicates that the CMC is a complex number).

**Table 3:** CMC values between FP-STA estimated using IMU and FP-STA measured using 3DMA.

		CMC					
		Preferred		Reduced		Lowest	
		Early-SLS Phase	Late-SLS Phase	Early-SLS Phase	Late-SLS Phase	Early-SLS Phase	Late-SLS Phase
Participant 1	Trial 1	0.70	0.96	0.30	0.59	NaN	NaN
	Trial 2	0.56	0.90	NaN	0.89	NaN	0.69
Participant 2	Trial 1	NaN	0.35	NaN	NaN	0.89	NaN
	Trial 2	NaN	0.56	NaN	0.72	NaN	NaN
Participant 3	Trial 1	NaN	NaN	NaN	NaN	NaN	NaN
	Trial 2	NaN	NaN	NaN	NaN	NaN	NaN

FP-SPA: frontal plane shank tilt angle; CMC: coefficient of multiple correlation; Preferred: preferred step rate (115 steps/min); Reduced: reduced step rate (100 steps/min); Lowest: lowest step rate (85 steps/min); Early-SLS phase: early phase of single-limb support; Late-SLS phase: late phase of single-limb support; NaN: not a number (indicates that the CMC is a complex number).

## Data Availability

The original data presented in this study are openly available at the institutional repository of Yamagata Prefectural University of Health Sciences: https://yachts.repo.nii.ac.jp/records/2000074, accessed on 13 September 2025.
